# Prevalence, toxigenic potential and antimicrobial susceptibility profile of *Staphylococcus* isolated from ready-to-eat meats

**DOI:** 10.14202/vetworld.2018.1214-1221

**Published:** 2018-09-05

**Authors:** Chinwe E. Okoli, Emmanuel Okechukwu Njoga, Simon I. Enem, Enid E. Godwin, John A. Nwanta, Kennedy F. Chah

**Affiliations:** 1 Department of Veterinary Public Health and Preventive Medicine, University of Abuja, Nigeria; 2 Department of Veterinary Public Health and Preventive Medicine, University of Nigeria, Nsukka, Enugu State, Nigeria; 3 Department of Veterinary Pathology and Microbiology, University of Nigeria, Nsukka, Enugu State, Nigeria

**Keywords:** antibiotic resistance, food safety, Nigeria, polymerase chain reaction, ready-to-eat meats, *Staphylococcus*

## Abstract

**Aim:**

An epidemiological surveillance for *Staphylococci* contamination of ready-to-eat (RTE) meats from Enugu State, Nigeria, was carried out to determine the prevalence, species distribution, toxigenic potential and antimicrobial susceptibility profile of the organisms and hence the microbiological and toxicological safety of the meats.

**Materials and Methods:**

Isolation and phenotypic *Staphylococcus* detection were done according to standard microbiological methods. Phenotypic resistance to 17 commonly used antimicrobial agents was determined by disc diffusion method. Molecular characterization of the isolates to species level and detection of selected toxigenic and antimicrobial-resistance genes were done by PCR methods.

**Results:**

Twenty-four (9.4%) of the 255 meat samples investigated were contaminated with *Staphylococcus* species. Twenty-four *Staphylococcus* isolates belonging to six species of coagulase-negative *Staphylococcus* (CoNS) were identified. Four (16.7%) isolates harbored genes coding for exfoliative toxin-A. Ten (41.7%) isolates were multidrug resistant, while *mecA*, *tetK*, *mphC*, *ermT* and *ermC* were the antimicrobial-resistance genes detected in the isolates. Meat samples sourced from motor parks (16.7%) and open markets (8.5%) were the most contaminated.

**Conclusion:**

9.4% of RTE meats sampled were contaminated with toxigenic and multidrug resistance CoNS. Beef was the most contaminated RTE meat type and harbored all the toxigenic and most of the antibiotic-resistant genes detected. Meat samples from motor parks had the highest staphylococcal contamination (16.7%), while those from mechanic village had the least (2.4%). Majority (79.2%) of the isolates were not susceptible to fusidic acid but none exhibited antimicrobial-resistance to chloramphenicol, ciprofloxacin, linezolid or teicoplanin. Food safety authorities in the study area should work proactively to massively improve the hygienic practices of meat vendors; in order to limit staphylococcal contamination of RTE meats and the associated public health problems.

## Introduction

*Staphylococcus* species are bacteria of veterinary and medical importance, capable of causing severe diseases in both humans and animals. *Staphylococci* are Gram-positive organisms, classified as coagulase-positive *Staphylococcus* (CoPS) or coagulase-negative *Staphylococcus* (CoNS), depending on their ability to produce coagulase enzyme [1,2]. Important members of the CoPS group are *Staphylococcus aureus* and *Staphylococcus pseudintermedius* because they are pathogenic and are often implicated in a wide range of disease conditions in animals and humans [2]. While CoNS are rarely pathogenic [2], they may cause opportunistic infections which sometimes degenerate to severe diseases in immune-compromised individuals. In addition, CoNS organisms may develop resistance to antimicrobial drugs due to the frequent exposure to low doses of the drugs as a result of imprudent use of antimicrobials in medical practice or animal agriculture [3]. Antimicrobial-resistant CoNS are reservoirs of antimicrobial resistance genes [1,2], transferrable to zoonotic or pathogenic organisms or to humans, through consumption of contaminated foods of animal origin [4-7], such as ready-to-eat (RTE) meats.

Ready-to-eat (RTE) meats refer to adequately prepared edible tissues of food animals that can be eaten without additional preparation to achieve food safety other than reheating. RTE meat types commonly sold in Nigeria are beef (*suya, balangu, kilishi* and *kundi*), pork, chicken andgoat meat (chevon). In most African settings, RTE meats are traditionally prepared by roasting and spicing of boneless portions of edible tissues of food animals, which may then be eaten alone or in combination with onions, herbs or vegetables. In Nigeria, RTE meats are very popular, especially among low- and medium-income earners, due to its low cost, appealing flavor and tantalizing taste [8]. The popularity of RTE meats in Nigeria is evident in the fact that the meats are sold along streets, served during ceremonies and parties, as well as in restaurants and fast food outlets, even those patronized by the elites.

Despite the popularity of RTE meats in Nigeria, the meats may be prone to microbial contamination due to some predisposing factors. High protein and water contents of the meats make them very susceptible to microbial contamination and multiplication [9], especially in the Tropics where climatic conditions favor the persistence and proliferation of most microbial pathogens [4-7]. The presence of microbial contaminants, especially *Staphylococcus* species, in RTE meats has generated a lot of public health and food safety concerns globally [10,11], due to their enormous pathogenic and zoonotic potentials [12] and the ease of transmission of the organisms to humans through consumption of the contaminated meat [13].

When organisms causing foodborne diseases (FBDs) such as *Staphylococcus* species are consumed through contaminated RTE meat; the onset of signs and symptoms of the disease such as abdominal discomfort, nausea, vomiting, diarrhea or even dysentery may follow shortly or may be delayed. Development of the signs and symptoms is however dependent on the virulence and pathogenic potentials of the ingested organisms, as well as the immune status of the individual [14]. When the contaminating microbes are not pathogenic or zoonotic, as is the case in most CoNS infections, the risk of possible transfer of antimicrobial resistant genes (if present in the microbial contaminants) to pathogenic or zoonotic pathogens in the gastrointestinal tract [3], following consumption of the contaminated meat still subsists [4-7].

Although most RTE meat vendors in Nigeria usually convey their meats in mobile carts or display them in wooden or glass cabinets to protect the meats from environmental contamination, there are indications that these meats may be contaminated to unsafe levels; with varying amounts of *Staphylococcus* and other pathogenic microbes [8], which may give rise to FBD outbreaks. Traditional methods of meat processing which may involve the use of crude instruments of unproven microbial quality [15], use of contaminated water during meat processing [16], and hawking or open-air display of meat to attract prospective buyers [17,18] as well as poor hygienic practices of RTE meat vendors [18] are leading causes of microbial contamination of RTE meats.

The ability of *Staphylococcus* organisms to grow on salty environments such as RTE meats [18] and capability of some of the species to elaborate toxins (enterotoxins and exfoliatins), through their toxin-producing genes [19] make the genus very important from food safety and public health points of view. *Staphylococcus* species are among the leading causes of food-related gastroenteritis or enterotoxemia [20]. The onset of symptoms in SFDs or intoxications is usually rapid and in many cases acute, depending on the susceptibility (immune status) of the individual involved, microbial load of the consumed meat and toxigenic potentials of the contaminants [19].

SFDs or intoxications (staphyloenterotoxicosis or staphyloenterotoxemia) are associated with enormous economic losses due to costs accruing from medical treatment, recall of contaminated food products, decreased productivity during the time of bedridden and reduced quality of life. It is estimated that *Staphylococcus* species cause about 241,000 illnesses per year in the USA [21]. Each case of SFD in some parts of the US costs about $695 in medical treatment and hence an estimated loss of 168 million dollars annually in medical treatment to SFD in the United States alone [21].

Despite the uncertainty associated with the microbial safety of RTE meats, the meat business continues to thrive in Nigeria. There is a paucity of information on the *Staphylococcus* contamination of RTE meats in Enugu State, Nigeria. The health risks posed by staphylococcal contamination of RTE meats to the meat consumers in the study area may be significant and need to be investigated. Epidemiological information on staphylococci contamination of RTE meats in the study area is essential for the provision of baseline data to guide public health intervention programs and empiric treatments during suspected cases of SFDs or intoxications.

The study was therefore conducted to determine the prevalence, toxigenic potential and antimicrobial susceptibility profile of *Staphylococcus* species from RTE meats in Enugu State, Nigeria. The study also ascertained the species distribution of *Staphylococcus* organisms recovered from RTE meats and also determined the significance of sampling locations on *Staphylococcus* contamination of RTE meats in the study area.

## Materials and Methods

### Ethical approval

Ethical approval is not applicable to this study as the researchers did not rear animals or sacrifice them to obtain the RTE meats used in this study. The meat samples were purchased from the vendors in the study area.

### Study area

The study was conducted in Enugu State, Southeast Nigeria. Enugu State has map coordinates of latitude 6°25´ North and longitude 7°27´ East and a population of about 5 million [3]. Most residents of the state are engaged in civil service, crop farming or buying and selling business; but small- and medium-scale livestock and poultry production, as an alternative source of income is widely practiced. The state has operational slaughterhouses where meat vendors source processed meat, but the preparation of RTE meats is usually done in their homes or at the business outfits. The state has some standard restaurants and fast food outlets, but there are lots of people hawking and selling RTE meats on the streets.

### Meat sample collection

A total of 255 roasted and spiced RTE meat samples, comprising 105 beef, 72 pork, 61 chicken parts and 17 goat meats (chevon) were purchased from vendors across different sampling locations (motor parks, mechanic village and open markets) in the study area. The sampling locations were purposively selected for the study because of high activities of RTE meat vendors in the chosen locations. However, simple random sampling method was used to select vendors sampled. About 50 g of each type of RTE meat vended was then purchased from the selected vendors. Sample collection visits were made forth nightly for 6 months and the selected vendors were sampled once per visit. The meat samples were separately packaged in sterile plastic bags and transported to the Veterinary Public Health Laboratory, University of Nigeria, Nsukka, for *Staphylococcus* isolation.

### Isolation and characterization of *Staphylococcus* species

Isolation, phenotypic and biochemical characterization of *Staphylococcus* spp. from the meat samples were performed following standard microbiological protocols [22] with minor modifications. Briefly, about 20 g of the outer surfaces of the meat were ascetically scrapped off, macerated and pre-enriched in the nutrient broth for 24 h at 37°C. A loop full of the pre-enriched samples was then streaked on mannitol salt agar (Oxoid, Basingstoke) and incubated at 37°C for 24 - 48 h. Three pink colonies were randomly selected from plates that produced growth and subculture on nutrient agar (Oxoid, Basingstoke) to purify the colonies. Representatives of the purified colonies, appearing cream on nutrient agar, were further subjected to Gram-staining and catalase test. Gram-positive cocci in bunches that produced vigorous bubbles on emulsification with 3% H_2_O_2_ were stocked on nutrient agar slants supplemented with sodium chloride for molecular characterization.

Molecular characterization of the stock *Staphylococcus* isolates to species level was done by polymerase chain reaction (PCR) amplification of *nuc* and *spa* genes according to the method and working conditions of Gomez-Sanz *et al*. [23]. Sequencing of *sodA* and 16S rDNA genes were performed according to the method and specifications of Poyart *et al*. [24]. Detection of genes coding for toxic shock syndrome (TSST-1) and exfoliative toxins A, B, and D (*eta*, *etb* and *etd* respectively) was done by PCR amplification of the respective genes according to the method described by Hwang and other workers [25].

Phenotypic resistance to 17 commonly used antimicrobial agents was performed using the disc diffusion method according to the specifications of the Clinical and Laboratory Standards Institute [26]. Antimicrobial-resistant isolates were then investigated for the presence of genes coding for resistance to methicillin/oxacillin (*mecA*), aminoglycosides (*aph(2)-aac(6), ant(4)*, and *aph(3)-III*), erythromycin (*ermA, ermB, ermC, ermT, mphC, msrA* and *msrB*), and tetracyclines (*tet(M), tet(O), tet(K)* and *tet(L*)) by PCR amplification of their specific oligonucleotide primers, as earlier described by Gomez-Sanz *et al*. [27].

### Statistical analysis

Chi-square statistic was used to determine whether there was significant association between staphylococcal contamination and meat types and sampling locations. The statistical tests were performed using IBM^®^ SPSS statistics version 23 (SPSS Inc., Chicago, Illinois, USA). Significance was accepted at p≤0.05.

## Results

### Prevalence of *Staphylococcus* according to meat types

*Staphylococcus* species were isolated from 24 (9.4%) of the 255 RTE meat samples investigated. Beef was the most contaminated (16.2%) RTE meat type while pork was the least with 4.2% prevalence (Table-1). A statistically significant association (p≤0.05) between *Staphylococcus* contamination and meat types was found at p = 0.022 (Table-1).

**Table-1:** *Staphylococcus* contamination of ready-to-eat meats according to meat types.

Meat types	Number sampled	Number (%) of contaminated samples	*χ*^2^-value	p-value
Beef (Suya)	105	17 (16.2)	9.675	0.022
Pork	72	3 (4.2)		
Chicken	61	3 (4.9)		
Goat meat (chevon)	17	1 (5.9)		
Total	255	24 (9.4)		

### Prevalence of *Staphylococcus* according to sampling locations

RTE meats sourced from motor parks and open markets were the most contaminated (Table-2). Meat samples from motor parks had the highest staphylococcal contamination (16.7%), while those from mechanic village had the least (2.4%). Statistically significant association (p≤0.05) was also found between *Staphylococcus* contamination and sampling sites at p = 0.043 (Table-2).

**Table-2:** *Staphylococcus* contamination of ready-to-eat meats according to sampling locations.

Sampling locations	Number sampled	Number (%) of contaminated samples	*χ*^2^-value	p-value
Open market	153	13 (8.5)	6.289	0.043
Motor park	60	10 (16.7)		
Mechanic village	42	1 (2.4)		
Total	255	24 (9.4)		

### Species of *Staphylococcus contaminants* isolated from RTE meats

Table-3 shows the species of *Staphylococcus* isolated from RTE meats. Twenty-four CoNS belonging to 6 different species, namely *Staphylococcus sciuri, Staphylococcus lentus, Staphylococcus saprophyticus, Staphylococcus carnosus, Staphylococcus piscifermentans* and *Staphylococcus epidermidis* were identified. All the six identified *Staphylococcus* species were present in beef.

**Table-3:** Distribution of *Staphylococcus species* (n=24) isolated from RTE meats from Enugu State, Nigeria.

*Staphylococcus* species identified	Total number (%) isolated
*S. sciuri*	13 (54.1)
*S. lentus*	4 (16.7)
*S. saprophyticus*	3 (12.5)
*S. carnosus*	2 (8.3)
*S. piscifermentans*	1 (4.2)
*S. epidermidis*	1 (4.2)

*S. sciuri=Staphylococcus sciuri, S. lentus=Staphylococcus lentus, S. saprophyticus=Staphylococcus saprophyticus, S. carnosus=Staphylococcus carnosus, S. piscifermentans=Staphylococcus piscifermentans, S. epidermidis=Staphylococcus epidermidis,* RTE=Ready-to-eat

### Toxigenic potential of the *Staphylococcus* isolates

Of the 24 *Staphylococcus* strains isolated, four (16.7%) harbored the virulence gene, exfoliative toxin A (*eta*), responsible for exfoliative toxin production. The four *eta*-positive strains were all *S. sciuri* isolated from beef. None of the *Staphylococcus* species harbored gene coding for exfoliative toxin B, exfoliative toxin C, TSST-1, Panton-Valentine leukocidin, or enterotoxin production.

### Antimicrobial resistance profile of the *Staphylococcus* species

The result of the antimicrobial susceptibility profile of the isolates is presented in Table-4. Nineteen (79.2%) of the staphylococci isolates were not susceptible to fusidic acid, while none of the isolates exhibited antimicrobial resistance to chloramphenicol, ciprofloxacin, linezolid or teicoplanin (Table-4).

**Table-4:** Antimicrobial susceptibility profile of *Staphylococcus* species (n=24) isolated from ready-to-eat meats.

Antimicrobial agents (ug)	Number (%) of resistant isolates
Fusidic acid (10)	19 (79.2)
Cefoxitin (30)	6 (25)
Oxacillin (10)	6 (25)
Tetracycline (30)	6 (25)
Erythromycin (30)	5 (20.8)
Lincomycin (10)	5 (20.8)
Vancomycin (30)	3 (12.5)
Mupirocin (5)	2 (8.3)
Sulfamethoxazole/trimethoprim (25)	2 (8.3)
Gentamicin (10)	1 (4.2)
Kanamycin (30)	1 (4.2)
Streptomycin (30)	1 (4.2)
Tobramycin (10)	1 (4.2)
Chloramphenicol (10)	0 (0)
Ciprofloxacin (5)	0 (0)
Linezolid (10)	0 (0)
Teicoplanin (5)	0 (0)

Of the 24 staphylococcal isolates identified, 10 (41.7%) were multidrug resistant. They exhibited resistance to three or more different classes of antimicrobials (Table-5). A total of 12 resistance patterns or phenotypes were recorded for the staphylococcal isolates with fusidic acid being involved in almost all the patterns (Table-5). Resistance genes detected in the isolates were *mecA (6*, 25%), *tetK (6*, 25%), *mphC* (3, 12.5%), *ermT (*2, 8.3%) and *ermC* (1, 4.2%). Two *tetK* genes and one *mecA* gene were detected in isolates from chicken and pork respectively, while the rest of the genes were found in isolates from beef (Table-6).

**Table-5:** Antimicrobial resistance patterns of *Staphylococcus* species (n=24) isolated from ready-to-eat meats.

Antimicrobial resistance phenotypes	Number (%) of isolate exhibiting the pattern
FA	7 (29.1)
TE	2 (8.3)
FA-TE	2 (8.3)
FA-VA	1 (4.2)
FA-MUP	1 (4.2)
TE-SXT	1 (4.2)
FA-CC-E	3 (12.5)
FA-OX-FOX	3 (12.5)
FA-TE-OX-FOX	1 (4.2)
FA-CC-VA-ER-TE	1 (4.2)
FA-CC-VA-ER-OX-FOX	1 (4.2)
S-SXT-KA-GE-TB-MUP	1 (4.2)

FA=Fusidic acid, FOX=Cefoxitin, OX=Oxacillin, TE=Tetracycline, CC=Clindamycin, ER=Erythromycin, LIN=Lincomycin, VA=Vancomycin, MUP=Mupirocin, SXT=Sulfamethoxazole/trimethoprim, GE=Gentamicin, KA=Kanamycin, S=Streptomycin, TB=Tobramycin

**Table-6:** Antimicrobial resistance genes detected from staphylococci isolated from different ready-to-eat meats.

Meat types	Strain ID	*Staphylococcus* species	Resistance phenotypes	Resistance genes detected
Beef (suya)	S_1_	*S. sciuri*	FA-OX-FOX	*mecA*
	S_6_	*S. sciuri*	FA-OX-FOX	*mecA*
	S_11_	*S. sciuri*	FA	*ermC*
	S_12_	*S. sciuri*	FA-OX-FOX	*mecA*
	S_15_	*S. sciuri*	FA	ND
	S_61_	*S. piscifermentans*	TE	*tetK*
	S_67_	*S. lentus*	FA-E-CC	*mphC*
	S_68_	*S. sciuri*	FA	ND
	S_69_	*S. epidermidis*	TE-SXT	*tetK*
	S_70_	*S. sciuri*	TE-FA-OX-FOX	*tetK, mecA*
	S_71_	*S. sciuri*	TE-FA	*tetK*
	S_72_	*S. sciuri*	FA	ND
	S_73_	*S. sciuri*	FA-OX-FOX-MUP	*mecA*
	S_74_	*S. lentus*	E-CC-FA	*mphC, ermT*
	S_75_	*S. lentus*	E-CC-FA	*mphC, ermT*
	S_80_	*S. saprophyticus*	FA	ND
	S_81_	*S. carnosus*	FA	ND
Pork	P_4_	*S. sciuri*	S-SXT-KA-GEN-TOB-MUP	ND
	P_23_	*S. saprophyticus*	FA	ND
	P_28_	*S. saprophyticus*	ER-CC-VA-FA-OX-FOX	*mecA*
Chicken	C_16_	*S. sciuri*	VA-FA	ND
	C_39_	*S. lentus*	ER-CC-VA-TE-FA	*TetK*
	C_13_	*S. carnosus*	TE	*TetK*
Goat meat	G_5_	*S. sciuri*	FA	ND

FA=Fusidic acid, FOX=Cefoxitin, OX=Oxacillin, TE=Tetracycline, CC=Clindamycin, ER=Erythromycin, LIN=Lincomycin, VA=Vancomycin, MUP=Mupirocin, SXT=Sulfamethoxazole/trimethoprim, GE=Gentamicin, KA=Kanamycin, S=Streptomycin, TB=Tobramycin, ND=Not detected, *S. sciuri=Staphylococcus sciuri, S. lentus=Staphylococcus lentus, S. saprophyticus=Staphylococcus saprophyticus, S. carnosus=Staphylococcus carnosus, S. piscifermentans=Staphylococcus piscifermentans, S. epidermidis=Staphylococcus epidermidis*

## Discussion

The 9.4% overall prevalence of *Staphylococcus* contamination of vended RTE meats revealed in this study is significant from food safety and public health points of view. The overall prevalence is lower than 28% prevalence reported by Mashak *et al*. [20] in Teheran Province, Iran, but higher than 3.2% reported in South Africa [14]. The disparity in the findings may be attributed to differences in the method of meat processing employed and overall hygienic practices of the meat vendors. Crude or traditional method of meat processing, involving the use of contaminated or obsolete meat processing equipment, makes meat contamination with microbes, especially *Staphylococcus* organisms, most probable since the organisms are ubiquitous in nature [28]. Environmental conditions such as the dusty streets or roads, in which some street meat vendors worked in the study area, may have favored contamination of RTE meats with *Staphylococcus* and hence the higher prevalence being reported. Also, exposure of RTE meats to open air along the streets to entice buyers, makes microbial contamination of the meats inevitable. In addition, poor hygienic practices of the meat vendors, especially during sale or packaging of already prepared meats, could facilitate staphylococcal contamination of the meats since *Staphylococcus* is a normal human flora.

Furthermore, ambient temperature variations across different regions of the world may impact of the survival and multiplication of *Staphylococcus* meat contaminants. Nigeria with an average ambient temperature of about 37°C is ideal for multiplication and survival of most microbial pathogens, hence increased chances of microbial contamination of street vended RTE meats. In addition to the temperature factor, inadequate municipal water supply, which encourages the use of other water sources of unproven microbial quality, may have contributed to the high prevalence of *Staphylococcus* contamination recorded in this study.

The high rate of *Staphylococcus* contamination of RTE meats recorded in motor parks and open markets may be due to heavy human and vehicular traffics which may facilitate microbial contamination of the meat. Senait and Moorty [29] reported that street hawking or open-air display of RTE meats in public places increases the bacterial load of the meats. This is because most of the bacteria contaminants are swept across by wind current and contaminated dust stirred by increased human or vehicular traffics. In addition, increased human activities and traffics at packs and open markets may increase the rate of discharge of *Staphylococcus* (which are known normal flora in humans) for contamination of poorly displayed foods of animal origin.

Sometimes, contamination of RTE meats with staphylococci may be due to poor post-preparation handling of the meats. Post-preparation contamination of RTE meats is a consequence of poor personal hygienic practices of the vendors. *Staphylococcus* species have been isolated from the body parts (nose, hands, fingertips, hairs and skin) of healthy individuals [27,30,]; hence, the vendors could serve as sources of the contamination. Post-preparation microbial contamination of RTE meat is a significant public health hazard because the vendors usually do not have the facilities needed to reheat the meat to depopulate the microbial contaminants. Besides, not all buyers are patient enough to wait for the reheating when the facilities or reheating-services are available. In addition, reheating, when possible, may not inactivate all microbial toxins, especially heat-stable toxins, if present in the meat.

The preponderance of the *Staphylococcus* contaminants in beef widely consumed in Nigeria [30] is disheartening in view of the toxigenic and antimicrobial resistance profiles of the contaminants. High rate of contamination of beef could be attributed to unwholesome slaughter or beef processing practices in some slaughterhouses in the study area [31,32]. The practice of flaying or evisceration of cattle carcass on bare floor or immersion of processed meat in water of unproven microbial quality, to make it appear bigger, predisposes meat to *Staphylococcus* contamination [33]. The use of contaminated equipments for bleeding, flaying, evisceration or transportation of processed meat also enhances staphylococci contamination. Additionally, poor evisceration practices in which the gut contents are allowed to come in contact with the meat makes contamination of the meat with gut microbes certain [33].

Although proper cooking (heating at 80 - 100°C for 30 min) may be sufficient to kill-off most bacterial contaminants in meats [3], some people do not engage in proper meat cooking due to fear of loss of volatile nutrients in the meat during cooking. This makes the risk of infection with microbial pathogens subsist, even after such cooking. In addition, cooking despite the heating temperature or duration may not eliminate all meat contaminants, especially heat-labile toxin.

Contamination of RTE meat with multidrug-resistant and toxin-producing *Staphylococcus* species poses enormous food safety concern. Although the *Staphylococcus* species identified in this study are rarely pathogenic, they can cause opportunistic infections in immunologically naive or immunocompromised individuals [1]. The multidrug-resistant organisms may serve as reservoirs of antimicrobial-resistant genes transferable to pathogenic *Staphylococcus* or other human pathogens for onward transmission to humans [3–7]. Consumption of the contaminated meat may predispose to gastrointestinal problems or other abnormalities in the event of extra-intestinal infections.

Detection of *eta* gene known to cause furuncles and carbuncles [1,2] and staphylococcal scalded skin syndrome in infants [2] from 16.7% of the *Staphylococci* isolates, shows that the meat consumers in the study area are at risk of the disease conditions. Isolation of antimicrobial resistant or toxigenic *Staphylococcus* species from foods of animal origin is of global concern [34,35] because these foods can be transported from one country to another, through international travels. This makes the global spread of antimicrobial-resistant or toxigenic organisms inevitable since the organisms do not respect international boundaries. Antibiotic-resistant *Staphylococcus* strains may cause diseases of high morbidity and mortality, lead to treatment failure and increase the cost of medical treatment and time of bedridden [36]. Exhibition of multidrug resistance to medical and veterinary essential antimicrobial agents, including cefoxitin, oxacillin and erythromycin as recorded in this study, is very worrisome because these drugs are still being heavily relied on, for the treatment of microbial diseases, especially in developing countries.

## Conclusion

We conclude that 9.4% of RTE meats investigated in Enugu State, Nigeria, were contaminated with toxigenic and multidrug-resistant CoNS species. Beef (16.2%) was the most contaminated RTE meat type while pork with prevalence of 4.2% recorded the least contamination. Beef harbored all the toxigenic genes and most of the antibiotic-resistant genes detected. Meat samples sourced from motor parks (16.7%) and open markets (8.5%) were the most contaminated. Most (79.2%) of the staphylococci isolates were not susceptible to fusidic acid but none of the isolates exhibited antimicrobial resistance to chloramphenicol, ciprofloxacin, linezolid or teicoplanin. Resistance genes detected in the isolates were *mecA*, *tetK*, mphC, *ermT* and *ermC*. These findings are of great public health and food safety concerns because RTE meats are usually consumed with little or no further preparation in the study area. Furthermore, CoNS are known opportunistic pathogens in neonates, the elderly and immunocompromised individuals [1,2]. The organisms are also reservoirs of antimicrobial-resistance genes [2], transmissible to humans via the food chain [3-7]. The findings of this work is a wake-up call to food safety and regulatory agencies in the study area, to swing into actions aimed at improving the hygienic practices of RTE meat vendors, to mitigate possible epidemics of SFDs and tremendous loses associated with such public health problems.

## Authors’ Contributions

KFC conceived the work. KFC, CEO and JAN designed the experiments; CEO, EON, SIE and EEG collected samples and performed the experiments. EON drafted the manuscript and performed the statistical analysis. All authors read and approved the final manuscript.
